# Increased Incidence of *IKZF1* deletions and *IGH-CRLF2* translocations in B-ALL of Hispanic/Latino children—a novel health disparity

**DOI:** 10.1038/s41375-021-01133-4

**Published:** 2021-02-02

**Authors:** Gordana Raca, Hisham Abdel-Azim, Feng Yue, James Broach, Jonathon L. Payne, Mark E. Reeves, Chandrika Gowda, Joseph Schramm, Dhimant Desai, Elanora Dovat, Tommy Hu, Arthur S. Berg, Deepa Bhojwani, Kimberly J. Payne, Sinisa Dovat

**Affiliations:** 1 Childrens Hospital Los Angeles, University of Southern California Keck School of Medicine, Los Angeles, CA USA; 2 Pennsylvania State University College of Medicine, Hershey, PA USA; 3 Loma Linda University School of Medicine, Loma Linda, CA USA

**Keywords:** Cancer genetics, Risk factors, Oncogenes

## To the Editor:

Hispanic/Latino (H/L) children and adolescents are 1.2–1.75 times more likely to develop acute lymphoblastic leukemia (ALL) than Non-Hispanic Whites (NHW) [1]. Once they develop ALL, H/L children have a 40% higher death-rate than NHW, after correcting for socioeconomic factors [2]. Although H/L children with B-ALL have a worse prognosis than non-H/L children, the biological basis for this health disparity is largely unknown. Single nucleotide polymorphisms (SNPs) in *ARID5B* and *GATA3* that are associated with predisposition to B-ALL and/or poor prognosis are more frequent among H/Ls [3, 4]. However, the major drivers of B-ALL through which these SNPs might contribute to health disparities have not been defined.

A previous study of children with high-risk B-ALL showed increased incidence of *CRLF2* gene rearrangement in H/L children as compared to the non-H/L population. *CRLF2* gene rearrangement was also associated with deletion of the *IKZF1* tumor suppressor [5]. A study of adult H/L patients with B-ALL showed increased incidence of Ph-like B-ALL that was associated with *CRLF2* rearrangement and *IKZF1* deletion [6]. Both studies were limited to subsets of B-ALL patients with high-risk features. Thus, the question of whether *CRLF2* gene rearrangement and/or *IKZF1* deletion provide a biological basis for the overall health disparity in pediatric B-ALL for H/L children remains unanswered. Here, we address this question by performing a single-center, unbiased analysis to determine and compare the incidence of *CRLF2* rearrangement and *IKZF1* deletion in H/L vs. non-H/L children with B-ALL.

We analyzed clinical and molecular data [7] from 239 pediatric B-ALL patients treated at Childrens Hospital Los Angeles between 3/2016 and 7/2019 (See Supplemental Materials). Of 239 patients diagnosed with B-ALL, 164 self-reported as H/L and 75 were classified as non-H/L (Table 1). *CRLF2* rearrangements include two types of genetic alterations: *IGH-CRLF2* translocation, where the immunoglobulin heavy chain locus (*IGH*) is translocated to *CRLF2* [8]; and *P2RY8-CRLF2* fusion, where the PAR1 deletion juxtaposes the noncoding exon of *P2RY8* to *CRLF2* [9]. Analysis of each of these genetic alterations separately, showed significantly increased incidence of *IGH-CRLF2* translocation in the H/L vs. non-H/L groups, 19/164 (12%) vs. 2/75 (2.7%), *p* = 0.026. However, the incidence of *P2RY8-CRLF2* fusion was not significantly different between the two populations.

**Table 1:** Characteristics of B-ALL in Hispanic/Latino and Non-Hispanic/Latino children.

Characteristic (all patients)	Overall^a^ *N* = 239	Hispanic/Latino^a^ *N* = 164	Non-H/L^a^ *N* = 75	*p* value^b^
Age	6.0 (3.0, 12.0)	7.0 (3.0, 13.0)	5.0 (3.0,11.0)	0.3
Gender				0.4
Female	106 (44%)	76 (46%)	30 (40%)	
Male	133 (56%)	88 (54%)	45 (60%)	
*IKZF1 deletion*	59 (25%)	48 (29%)	11 (15%)	**0.016**
*CRLF2* translocation (all)	36 (15%)	28 (17%)	8 (11%)	0.2
*IGH-CRLF2*	21 (8.8%)	19 (12%)	2 (2.7%)	**0.026**
*P2RY8-CRLF2*^c^	15 (6.3%)	9 (5.5%)	6 (8.0%)	0.6
*IKZF1* & *CRLF2*	20 (8.4%)	20 (12%)	0 (0%)	**<0.001**
*IKZF1* & *IGH-CRLF2*	18 (7.5%)	18 (11%)	0 (0%)	**0.001**
*IKZF1* & *P2RY8-CRLF2*	2 (0.8%)	2 (1.2%)	0 (0%)	>0.9
*IKZF1* & no *IGH-CRLF2*	41 (17%)	30 (18%)	11 (15%)	0.6
*IGH-CRLF2* & no *IKZF1*	3 (1.3%)	1 (0.6%)	2 (2.7%)	0.2
*P2RY8-CRLF2* & no *IKZF1*	13 (5.4%)	7 (4.3%)	6 (8.0%)	0.2
Ph + ALL	12 (5.0%)	5 (3.0%)	7 (9.3%)	0.054

Children age ≥ 10 only				
Characteristic (Age ≥ 10)	Overall^a^ *N* = 83	Hispanic/Latino^a^ *N* = 59	Non-H/L^a^ *N* = 24	*p* value^b^
Age	15.00 (11.00, 17.00)	14.00 (11.50, 17.00)	15.00 (11.00,17.00)	0.8
Gender				>0.9
Female	34 (41%)	24 (41%)	10 (42%)	
Male	49 (59%)	35 (59%)	14 (58%)	
*IKZF1* deletion	40 (48%)	35 (59%)	5 (21%)	**0.002**
*CRLF2* translocation (all)	21 (25%)	19 (32%)	2 (8.3%)	**0.027**
*IGH-CRLF2*	18 (22%)	18 (31%)	0 (0%)	**0.001**
*P2RY8-CRLF2*	3 (3.6%)	1 (1.7%)	2 (8.3%)	0.2
*IKZF1* & *CRLF2*	19 (23%)	19 (32%)	0 (0%)	**<0.001**
*IKZF1* & *IGH-CRLF2*	18 (22%)	18 (31%)	0 (0%)	**0.001**
*IKZF1* & *P2RY8-CRLF2*	1 (1.2%)	1 (1.7%)	0 (0%)	>0.9
*IKZF1* & no *IGH-CRLF2*	22 (27%)	17 (29%)	5 (21%)	0.6
*IGH-CRLF2* & no *IKZF1*	0 (0%)	0 (0%)	0 (0%)	NA
*P2RY8-CRLF2* & no *IKZF1*	2 (2.4%)	0 (0%)	2 (8.3%)	0.081
Ph + ALL	4 (4.8%)	2 (3.4%)	2 (8.3%)	0.6

Bold values indicate statistical significance *p* < 0.05.

^a^Statistics presented: median (IQR); *n* (%).

^b^Statistical tests performed: Wilcoxon rank-sum test; Fisher’s exact test.

^c^The *P2RY8-CRLF2* translocation is more common in Down Syndrome B-ALL, the H/L cohort included three Down Syndrome cases (one *P2RY8-CRLF2* and two unknown genetics); the Other cohort included four Down Syndrome cases (three cases *P2RY8-CRLF2*, and one hyperploidy).

B-ALL in the H/L population showed a significantly higher incidence of *IKZF1* deletion as compared to non-H/Ls, 48/164 (29%) vs. 11/75 (15%), *p* = 0.016. These results suggest that *IKZF1* deletion is a novel biological determinant of the health disparity in pediatric B-ALL for H/L children.

Since the previous data suggested an association between *CRLF2* translocations and *IKZF1* deletion, we analyzed the incidence of patients with concomitant *IKZF1* deletion and *CRLF2* translocations. The concomitant *IKZF1* deletion with either type (*IGH-CRLF2* or *P2RY8-CRLF2*) of *CRLF2* translocation was strongly increased in the H/L vs. non-H/L population, 20/164 (12.0%) vs. 0/75 (0%), *p* < 0.0001. However, there was a strong bias in association of *IKZF1* deletion with a particular *CRLF2* translocation; the *IgH-CRLF2* translocation was ninefold increased over the *P2RY8-CRLF2* fusion (18/164 vs. 2/164) in patients with *IKZF1* deletion. As a consequence, *IKZF1* deletions concomitant with *IGH-CRLF2* translocation were strongly increased in the H/L vs. non-H/L population, 18/164 (11%) vs. 0/164 (0%), *p* = 0.001. A concomitant *IKZF1* deletion with *P2RY8-CRLF2* fusion was observed in only two patients, both in the H/L population.

These data demonstrate that the *IGH-CRLF2* translocation, the *IKZF1* deletion and the concomitant *IKZF1* deletion with *IGH-CRLF2* translocation are highly increased in B-ALL of children in the H/L population. The incidence of *P2RY8-CRLF2* fusion is not different between the two populations. *IKZF1* deletion and *IGH-CRLF2* translocation are each associated with poor prognosis [10–13] and both IKAROS and CRLF2 proteins regulate a large number of genes and/or pathways that promote leukemia progression and drug resistance [14, 15]. Thus, these data provide evidence of *IGH-CRLF2* translocation and *IKZF1* deletion as biological determinants of the health disparity in pediatric B-ALL for H/L patients and suggest a biological rationale for the inferior outcome of H/L children with this disease.

We analyzed whether the age of patients affects the incidence, and/or racial difference of the above genetic alterations in B-ALL. In children ≥10 yrs old (Table 1), the incidence of *IKZF1* deletion is 2.8-fold increased in the H/L vs. non-H/L population, 35/59 (59%) vs. 5/24 (21%), *p* = 0.002, with an odds ratio of 5.4. *IKZF1* deletion is highly increased in children ≥10 yrs (59%) vs. <10 yrs (12%) in the H/L population (Table S2), but not in the non-H/L group. In children ≥10 yrs, the incidence of *IGH-CRLF2* translocation was strongly increased in the H/L vs. non-H/L population, 18/59 (31%) vs. 0/24 (0%), *p* = 0.001. The incidence of *IGH-CRLF2* translocation is highly increased in children ≥10 yrs, 18/59 (31%) old vs. <10 yrs old, 1/105 (1%) in the H/L population, but not in the non-H/L group. All of the patients ≥10 yrs with *IGH-CRLF2* translocations in both the H/L and the non-H/L group also had concomitant *IKZF1* deletions. Thus, no patient ≥10 yrs had *IGH-CRLF2* translocation without concomitant *IKZF1* deletion. In contrast, in patients ≥10 yrs old, the *IKZF1* deletion without the presence of the *IGH-CRLF2* translocation was detected in 17/59 (29%) of the H/L population.

In children <10 yrs, neither *IGH-CRLF2* translocation, *IKZF1* deletion, nor the combination of these two genetic alterations showed significant difference in incidence between the H/L and non-H/L populations (Table S1).

When analysis included only Ph negative B-ALL (Table S2–S4), the incidence of *IKZF1* deletion was greater than threefold increased in the H/L vs. non-H/L population, 44/159 (28%) vs. 6/68 (8.8%), *p* = 0.001 and greater than fourfold increased in children ≥10 yrs old, 33/57 (58%) vs. 3/22 (14%), *p* < 0.001.

The results of our study provide a biological rationale for the worse prognosis of B-ALL in H/L children. Our unbiased, single-institution study identified highly increased incidence of *IGH-CRLF2* translocation, *IKZF1* deletion and concomitant *IGH-CRLF2* translocation with *IKZF1* deletion in B-ALL of H/L children. The approximate fourfold increased incidence in H/L children with B-ALL, makes *IGH-CRLF2* the single genetic alteration with the highest racial/ethnic pediatric cancer disparity. The very high overall incidence (29%), makes *IKZF1* deletion the most frequent genetic alteration that confers adverse prognosis in B-ALL in H/L children. However, the largest difference between H/L and non-H/L children was the presence of the concomitant *IKZF1* deletion with *IGH-CRLF2* translocation, which was detected in 11% of H/L children, but was not detected in any leukemia of non-H/L children.

**Fig. 1:**
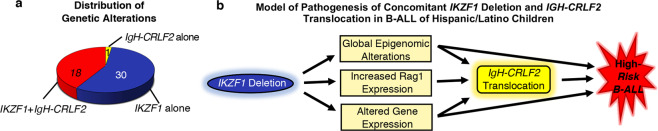
*IKZF1* Deletion *IGH-CRLF2* Translocation in CRLF2 B-ALL of Hispanic Children. **a** Relationship between *IKZF1* deletion and *IGH-CRLF2* translocation in B-ALL of Hispanic/Latino children. **b** Model of pathogenesis of concomitant *IKZF1* deletion and *IGH-CRLF2* translocation in B-ALL of Hispanic/Latino children.

The disparity in incidence of *IGH-CRLF2* translocation and *IKZF1* deletion in H/L children vs. non-H/Ls was very strong in children ≥10 yrs, but not in younger children. The most intriguing finding in our study was that over 94% (18/19) B-ALL in H/L children with *IGH-CRLF2* translocation had concomitant *IKZF1* deletion. In contrast, 30 H/L children with B-ALL had *IKZF1* deletion without concomitant *IGH-CRLF2* translocation. This raises the strong possibility that *IKZF1* deletion precedes *IGH-CRLF2* translocation, and/or that *IKZF1* deletion predisposes cells to *IGH-CRLF2* translocation in B-ALL of H/L children. IKAROS represses transcription of the RAG1 gene and increased expression of RAG1 due to *IKZF1* deletion, might play a role in this process (Fig. 1). The results of our study lead to two main questions: (1) What biological factors cause increased incidence of *IKZF1* deletion in H/L children; and (2) Does the presence of *IKZF1* deletion in the B-lineage make cells more susceptible to the *IGH-CRLF2* translocation, and if so, why is that susceptibility stronger in H/L children than in non-H/L populations. One *GATA3* SNP occurs at higher frequency in the H/L population and has been associated with increased susceptibility to *CRLF2* rearrangement and *IKZF1* deletion [3, 4]. However, functional studies to evaluate the potential role of *GATA3* or non-H/L biological factors in *CRLF2* and *IKZF1* alterations have not been performed. Answering these questions will help in understanding the pathogenesis of pediatric B-ALL and the biological basis of the B-ALL health disparity in H/L children.

In summary, the presented data demonstrate that *IGH-CRLF2* translocation and *IKZF1* deletion provide a biological basis for the health disparity in pediatric B-ALL for H/L children and a strong biological rationale for the higher death-rate they experience due to B-ALL. Our study suggests that, in addition to reducing socioeconomic inequities, the following changes in clinical practice would improve the prognosis of H/L children with B-ALL: (1) due to the high incidence of *IGH-CRLF2* translocation and *IKZF1* deletion, every child of H/L background with B-ALL should be tested specifically for the presence of both of these genetic alterations; and (2) novel treatment strategies that restore IKAROS function while targeting CRLF2 signaling pathways (e.g., JAK/STAT or PI3K/AKT/mTOR), should be developed and clinically tested to reduce the health disparity in pediatric B-ALL.

## Supplementary information

Supplemental Materials
