# Supplementary material for: Increased Incidence of IKZF1 deletions and IGH-CRLF2 translocations in B-ALL of Hispanic/Latino children—a novel health disparity
Source: Leukemia. 2021 Feb 2;35(8):2399–402. doi: 10.1038/s41375-021-01133-4 (PMC8324481; doi:10.1038/s41375-021-01133-4)
Supplement: Supplementary file 1 — Supplemental Materials [file 41375_2021_1133_MOESM1_ESM.pdf]

Supplemental Materials

Increased Incidence of *IKZF1* Deletions and *IGH-CRLF2* Translocations in B-ALL of Hispanic/Latino children – a Novel Health Disparity

Gordana Raca[1*], Hisham Abdel-Azim[1], Feng Yue[2], James Broach[2], Jonathon L. Payne[3,2], Mark E. Reeves[3], Chandrika Gowda[2], Joseph Schramm[2], Dhimant Desai[2], Elanora Dovat[2], Tommy Hu[2], Arthur S. Berg[2], Deepa Bhojwani[1], Kimberly J. Payne[3*] and Sinisa Dovat[2*]

[1]Children Hospital Los Angeles, University of Southern California Keck School of Medicine, USA.
[2]Pennsylvania State University College of Medicine, Hershey, PA, USA
[3]Loma Linda University School of Medicine, Loma Linda, CA, USA

***Corresponding Authors:**

Sinisa Dovat, M.D., Ph.D.
Pennsylvania State University College of Medicine,
Department of Pediatrics
500 University Drive
Hershey, PA 17033, USA
Telephone: 717-531-6012
E-mail: sdovat@pennstatehealth.psu.edu

Kimberly J. Payne, PhD
Loma Linda University
11085 Campus St.
First Floor Mortensen Hall
Loma Linda, CA 92350
Telephone: 909-558-1000 X81363
Email: kpayne@llu.edu

Gordana Raca, MD, PhD, FACMG
Childrens Hospital Los Angeles
4650 W. Sunset Blvd.
Los Angeles CA 90027
Telephone: 323-644-850
Email: graca@chla.usc.edu

Supplemental Materials

Supplemental Materials, Subjects and Methods:

Subjects.
Deidentified clinical and molecular data from 239 pediatric B-ALL patients treated at the Children's Hospital Los Angeles (CHLA) between 3/2016 and 7/2019 were analyzed in compliance with Institutional Review Board regulations. Of the 239 pediatric patients diagnosed with B-ALL at CHLA, 164 self-reported as H/L and 75 non-H/L. One additional patient with unknown ethnicity was excluded from this study. Three patients with B-lymphoblastic lymphoma were included (molecular studies were performed on tissue). Genetic ancestry was not assessed.

Methods. All testing was conducted as a part of the clinical care of children with newly diagnosed (N=201) or relapsed (N=38) leukemias. Testing for 98 of the cases included karyotype analysis, fluorescence in situ hybridization (FISH) testing for BCR/ABL1, ETV6/RUNX1, KMT2A rearrangements and trisomy 4 and 10, and Chromosomal microarray analysis (CMA); in 141 cases testing also included the OncoKids® NGS panel [1]. The presence of the IKZF1 deletion was detected by CMA analysis. CRLF2 expression was initially detected by flow-cytometry; in positive cases, CRLF2 rearrangements were confirmed and characterized by FISH, using CRLF2 and IGH break-apart probes.

Statistical analysis: Wilcoxon rank-sum test and the Fisher exact test were used to determine association between categorical variables.

Reference

1. Hiemenz MC, Ostrow DG, Busse TM, Buckley J, Maglinte DT, Bootwalla M, Done J, Ji J, Raca G, Ryutov A, Xu X, Zhen CJ, Conroy JM, Hazard FK, Deignan JL, Rogers BB, Treece AL, Parham DM, Gai X, Judkins AR, Triche TJ, Biegel JA. OncoKids: A Comprehensive Next-Generation Sequencing Panel for Pediatric Malignancies. J Mol Diagn. 2018;20(6):765-76. Epub 2018/08/24. doi: 10.1016/j.jmoldx.2018.06.009. PubMed PMID: 30138724.

Supplemental Tables

Characteristic	Overall[1] N = 156	H/L[1] N = 105	Non-H/L[1] N = 51	p-value[2]
**Age**	3.00 (2.00, 6.00)	4.00 (2.00, 6.00)	3.00 (2.00, 5.00)	0.4
**Gender**				0.2
Female	72 (46%)	52 (50%)	20 (39%)	
Male	84 (54%)	53 (50%)	31 (61%)	
***IKZF1* deletion**	19 (12%)	13 (12%)	6 (12%)	>0.9
***CRLF2* translocation**	15 (9.6%)	9 (8.6%)	6 (12%)	0.6
IGH-CRLF2	3 (1.9%)	1 (1.0%)	2 (3.9%)	0.2
P2RY8-CRLF2	12 (7.7%)	8 (7.6%)	4 (7.8%)	>0.9
IKZF1* & *CRLF2	1 (0.6%)	1 (1.0%)	0 (0%)	>0.9
IKZF1* & *IGH-CRLF2	0 (0%)	0 (0%)	0 (0%)	N/A
IKZF1* & *P2RY8-CRLF2	1 (0.6%)	1 (1.0%)	0 (0%)	>0.9
IKZF1* & no *IGH-CRLF2	19 (12%)	13 (12%)	6 (12%)	>0.9
IGH-CRLF2* & no *IKZF1	3 (1.9%)	1 (1.0%)	2 (3.9%)	0.2
P2RY8-CRLF2* & no *IKZF1	11 (7.1%)	7 (6.7%)	4 (7.8%)	0.8
**Ph+ leukemia**	8 (5.1%)	3 (2.9%)	5 (9.8%)	0.11

Supplementary Table S1: Children Age <10 Only

[1] Statistics presented: median (IQR); n (%)

[2] Statistical tests performed: Wilcoxon rank-sum test; Fisher's exact test

Characteristic	Overall[1] N = 227	Hispanic/Latino[1] N = 159	Non-H/L[1] N = 68	p-value[2]
**Age**	6.00 (3.00, 12.00)	7.00 (3.00, 12.00)	5.00 (2.8, 11.0)	0.3
**Gender**				0.7
Female	103 (45%)	74 (47%)	29 (43%)	
Male	124 (55%)	85 (53%)	39 (57%)	
***IKZF1* deletion**	50 (22%)	44 (28%)	6 (8.8%)	**0.001**
***CRLF2* translocation**	36 (16%)	28 (18%)	8 (12%)	0.3
IGH-CRLF2	21 (9.3%)	19 (12%)	2 (2.9%)	**0.043**
P2RY8-CRLF2 [3]	15 (6.6%)	9 (5.7%)	6 (8.8%)	0.4
IKZF1* & *CRLF2	20 (8.8%)	20 (13%)	0 (0%)	**<0.001**
IKZF1* & *IGH-CRLF2	18 (7.9%)	18 (11%)	0 (0%)	**0.002**
IKZF1* & *P2RY8-CRLF2	2 (0.9%)	2 (1.3%)	0 (0%)	>0.9
IKZF1* & no *IGH-CRLF2	32 (14%)	26 (16%)	6 (8.8%)	0.2
IGH-CRLF2* & no *IKZF1	3 (1.3%)	1 (0.6%)	2 (2.9%)	0.2
P2RY8-CRLF2* & no *IKZF1	13 (5.7%)	7 (4.4%)	6 (8.8%)	0.2

Supplementary Table S2: Children (all patients), Ph Negative B-ALL

[1] Statistics presented: median (IQR); n (%)

[2] Statistical tests performed: Wilcoxon rank-sum test; Fisher's exact test

[3] The *P2RY8-CRLF2* translocation is more common in Down Syndrome B-ALL, the H/L cohort included 3 Down Syndrome cases (one *P2RY8-CRLF2* and two unknown genetics); the Other cohort included 4 Down Syndrome cases (3 cases *P2RY8-CRLF2*, and one hyperploidy).

Characteristic	Overall[1] N = 79	Hispanic/Latino[1] N = 57	Non-H/L[1] N = 22	p-value[2]
**Age**	15.00 (11.00, 17.00)	14.00 (12.00, 17.00)	15.00 (11.00, 16.75)	0.7
**Gender**				0.8
Female	33 (42%)	23 (40%)	10 (45%)	
Male	46 (58%)	34 (60%)	12 (55%)	
***IKZF1* deletion**	36 (46%)	33 (58%)	3 (14%)	**<0.001**
***CRLF2* translocation**	21 (27%)	19 (33%)	2 (9.1%)	**0.045**
IGH-CRLF2	18 (23%)	18 (32%)	0 (0%)	**0.002**
P2RY8-CRLF2	3 (3.8%)	1 (1.8%)	2 (9.1%)	0.2
IKZF1* & *CRLF2	19 (24%)	19 (33%)	0 (0%)	**<0.001**
IKZF1* & *IGH-CRLF2	18 (23%)	18 (32%)	0 (0%)	**0.002**
IKZF1* & *P2RY8-CRLF2	1 (1.3%)	1 (1.8%)	0 (0%)	>0.9
IKZF1* & no *IGH-CRLF2	18 (23%)	15 (26%)	3 (14%)	0.4
IGH-CRLF2* & no *IKZF1	0 (0%)	0 (0%)	0 (0%)	N/A
P2RY8-CRLF2* & no *IKZF1	2 (2.5%)	0 (0%)	2 (9.1%)	0.075

Supplementary Table S3: Children Age ≥10 Only, Ph Negative B-ALL

[2] Statistics presented: median (IQR); n (%)

[2] Statistical tests performed: Wilcoxon rank-sum test; Fisher's exact test

**Characteristic**	**Overall**[1] N = 148	**Hispanic/Latino**[1] N = 102	**Non-H/L**[1] N = 46	**p-value**[2]
**Age**	3.00 (2.00, 6.00)	4.00 (2.00, 6.00)	3.00 (2.00, 5.00)	0.3
**Gender**				0.4
Female	70 (47%)	51 (50%)	19 (41%)	
Male	78 (53%)	51 (50%)	27 (59%)	
***IKZF1* deletion**	14 (9.5%)	11 (11%)	3 (6.5%)	0.6
***CRLF2* translocation**	15 (10%)	9 (8.8%)	6 (13%)	0.6
IGH-CRLF2	3 (2.0%)	1 (1.0%)	2 (4.3%)	0.2
P2RY8-CRLF2	12 (8.1%)	8 (7.8%)	4 (8.7%)	>0.9
IKZF1* & *CRLF2	1 (0.7%)	1 (1.0%)	0 (0%)	>0.9
IKZF1* & *IGH-CRLF2	0 (0%)	0 (0%)	0 (0%)	N/A
IKZF1* & *P2RY8-CRLF2	1 (0.7%)	1 (1.0%)	0 (0%)	>0.9
IKZF1* & no *IGH-CRLF2	14 (9.5%)	11 (11%)	3 (6.5%)	0.6
IGH-CRLF2* & no *IKZF1	3 (2.0%)	1 (1.0%)	2 (4.3%)	0.2
P2RY8-CRLF2* & no *IKZF1	11 (7.4%)	7 (6.9%)	4 (8.7%)	0.7

Supplementary Table S4: Children Age <10 Only, Ph Negative B-ALL

[1] Statistics presented: median (IQR); n (%)

[2] Statistical tests performed: Wilcoxon rank-sum test; Fisher's exact test
